# The Medical Society of the County of Erie

**Published:** 1892-02

**Authors:** Eli H. Long

**Affiliations:** Secretary


### THE MEDICAL SOCIETY OF THE COUNTY OF ERIE.

Reported by ELI H. LONG, M.D., Secretary.

The annual meeting of the Medical Society of the County of Erie, held January 12, 1892, was called to order at 10 o'clock A. M. by the President, Dr. E. C. W. O'Brien.

The minutes of the last regular meeting and two special meetings were read, and, upon motion of Dr. J. J. Walsh, adopted.

Dr. M. D. Mann moved that the record of censure passed upon Dr. Harrington be stricken from the minutes, and that all matter referring thereto, together with new evidence which he would present, be referred back to the Board of Censors to report at the next meeting of the society. The motion was seconded by Dr. Hopkins.

Dr. J. D. Bonnar moved to amend that the case be reopened before the society. Seconded by Dr. E. L. Bissell.

A discussion followed, participated in by Drs. Storck, Hayd, Potter, Harrington, Hopkins, Coakley, Bartlett, Cronyn, Abbott, and others, in course of which Dr. Harrington expressed deep regret for his uncomplimentary reference to the members of the society which had appeared in the public press. In consideration of this and other statements made in explanation by Dr. Harrington, it was moved by Dr. Wyckoff as a substitute motion that these be accepted as a sufficient apology, and that the whole matter be stricken from the records of the society. Carried.

The Secretary moved that the chair appoint a committee of five to nominate officers for the ensuing year, to report at the afternoon session. This was amended upon motion of Dr. Storck to the effect that when the proper order of business is reached, the society shall proceed to nominate and elect officers in open session. Carried. The Committee on Membership recommended the following-named physicians for election to membership: W. H. Baker, C. A. Schladermundt, Harriet E. Sheldon, Fred H. Powell, Chas. S. Meahl, E. H. Tweedy, Chas. H. Woodward, Chas. P. Clark, Lillian C. Randall, A. B. Kinsley, Lewis G. Smith, Irving W. Potter, Arthur B. Allen, Chas. J. Reynolds, John R. Gray, Francis L. Watkins, Frederick Preiss, and F. E. Hill.

Upon motion they were duly elected, and those present were introduced to the society.

Dr. Hubbell moved that after "Communications from Abroad," in the regular order of business, the programme of scientific papers be taken up. Carried.

The Committee on Membership reported the following applications for membership, to be acted upon at the next regular meeting: W. F. Beutler, J. M. Hewitt, A. J. Colton, M. V. Ball, J. J. Drake, L. A. Denton, Mary I. Denton, H. U. Williams, J. T. Harris, E. J. Gilray, B. C. Johnson, A. E. Collins, and A. G. Bennett.

The Treasurer presented his report, showing a balance in the treasury of $292.53, which, upon motion, was referred to an auditing committee, consisting of Drs. Cott, Hayd and Edward Clark. Upon their favorable report the Treasurer's accounts were passed.

The Board of Censors, through its chairman, Dr. E. Storck, presented the following report:

*To the Medical Society of the County of Erie:*

The Board of Censors at its last meeting directed its chairman to report as follows:

At the semi-annual meeting, in June, 1891, the society was informed that the board had notified Dr. Francisco Palmiere that he was practising in this city in violation of the State law, as he had no diploma registered in our County Clerk's office. It was also claimed by some of his countrymen, who appeared before your board, that he was not the rightful owner of the diploma that he possessed. The diploma was submitted to Dr. J. Cronyn, the President of the medical faculty of Niagara University, who declined to endorse it, for the reason that he was not convinced of the identity of the person. The applicant having refused to submit to a personal examination, the diploma was then submitted to the State Board of Medical Examiners for endorsement, and license applied for through his legal adviser. The State Board was informed of the foregoing statement of facts, and of the action taken thereon by your board.

Prof. Thomas, the Chief Examiner, has lately sent to your board a letter that he had received from the Italian Consul-General in New York, which reads as follows: "The foreign office in Rome has directed me to assure you that the diploma submitted to me has been inspected by the University of Naples; the same has been recognized as *genuine*." The Chief Examiner adds: "The letter explains itself. We now see no reason why the diploma of Dr. Palmiere should not be endorsed. Dr. Palmiere has complied with our requirements, the only question in his case being the genuineness of his diploma."

Though this decision settles the question satisfactorily to Dr. Palmiere, your board begs leave to differ from it in one respect. We hold that the genuineness of the diploma should not be the only criterion, but the identity of the person should be established beyond a doubt. This can only be done by a personal examination.

The case of Ferdinand Simon has finally been disposed of by the State Examiners. They rejected the documents he had submitted as being simply testimonials of service as hospital steward, therefore no license was granted. This case has become quite a *cause célèbre.* Medical journals east and west have taken notice of it. After a member of this society had been expelled for having been connected in business and practice with Simon, the board was informed that legal proceedings would be commenced against this society for its action, as Dr. Simon was in possession of a diploma of a legal medical college in Cincinnati, O. Your board, however, induced the District Attorney to bring the case again before Judge Seaver. At the trial the plea was brought up by the learned counsel for the defendant, who claimed that such a diploma was to be submitted to the State Board of Examiners. Further time was granted by the court. The diploma was examined by Dr. W. W. Potter, one of the State Examiners. It had been granted to Ferdinand Simon by the American Eclectic College, of Cincinnati, O., and is dated June 6, 1891, bearing the signatures of nine professors and the golden seal of the institution. It was obtained by Simon —so he informed your board—in one day. He passed a *pro forma* examination before one of the professors for which he had to pay a round fee, and $25.00 in addition for the diploma. This diploma was naturally rejected by our State Examiners, and no license granted. The result of this case has proven two satisfactory points: First, that your board has been fully justified in the prosecution of Simon and his partner; second, it has demonstrated the great advantage of having a State Licensing Board that will not issue a license to practise in this State on the mere presentation of a sheep-skin of a so-called medical college that claims to have a legal existence. The days of such institutions are numbered.

Of the other cases your board had brought before the Grand Jury: Horace Dewey and Ellen Crossley after they had been indicted decamped for a more genial climate. The District Attorney does not seem to be very anxious to look them up, neither are we.

The clairvoyant doctress that was not indicted on account of that rheumatic grand juror from a "rural deestrict," is still quacking in violation of the law. Her case, however, is in the hands of the District Attorney, who will again bring it before the grand jury if sufficient evidence can be furnished.

Dr. C. Zolotwitzki, a Russian physician on Clinton street, has been practising for some time in this city. He has been notified by the board that he is violating the medical law, not having effected any registry. The diploma he holds of a Russian medical college seems to be genuine, but so far he has not submitted the same to the State examiners. Unless he complies with the law, the Censors will ask the District Attorney to prosecute him, and he has been served with notice to that effect.

This completes the business of the Board of Censors for the year 1891. The chairman hopes that this will also be the last report he will be called upon to make. Having performed the duties and labors of this office—which have often been onerous and disagreeable—cheerfully and conscientiously, he now asks the society to relieve him from the task in the future. Recent unpleasant occurrences have made it doubly a burden. The Censors, as the legal executive officers of the society in carrying out the medical laws and the by-laws of the society, in bringing offenders to justice and discipline, necessarily have made personal enemies; but that the chairman, discharging his duties without fear or favor, should call down upon himself the wrath and displeasure of some of the prominent members of the medical profession and be persecuted and condemned by them, seems rather strange. The board, since he has been a member and chairman thereof (twelve years), has endeavored to elevate the standard of the medical profession and of medical education. Some twenty years ago he submitted a bill to the Legislature "to regulate the practice of medicine and surgery in this State;" it was passed by both Houses, but some prominent and, I may add, orthodox physicians of Albany prevailed upon Gov. Hoffman to veto it. A few years later the bill passed the Assembly, but was smothered in the committee box in the Senate. In 1880

we finally got the registry law in force, that made it a misdemeanor to practise without having a diploma or license duly registered. The censors of both schools of medicine in this county first carried this law into effect. The itinerant quack was driven out of the city or arrested ; the local irregulars were compelled to comply with the law or desist from practising. The people, more than the profession, received protection from the enforcement of this law. It was your Board of Censors that first instituted legal proceedings, by applying to the Attorney-General of the State for a writ against the bogus medical colleges that granted diplomas without having a legal charter. We argued the case before Gov. Cleveland, who declared their charters null and void. This action involved considerable expense to our society, but the result was worth the money.

The first action taken to secure the passage of a law "to create a State Board of Medical Examiners," was by a resolution of your board that passed this Society eight years ago ; further action thereon was taken, to which Dr. W. W. Potter, of this city, referred in his address as President of the State Medical Society last year as follows : "Year by year the note of warning has been sounded on the subject of separating the teaching and licensing powers. More definite shape, however, was given to the matter some seven or more years ago, when the Committee on Legislation of the Medical Society of the County of Erie reported a plan for the complete separation of the 'unfortunate, unwise and debasing copartnership of the educating and licensing powers.' The bill drawn by this committee was submitted to and approved by the State Medical Society and introduced in the Legislature ; it became a law June 5, 1890, and is now in full operation."

This is a résumé of our work done in the past. If the writer has contributed a good share thereto, and helped to secure these great results, which will prove beneficial to the medical profession and the people of this State alike ; if his efforts in this direction have met your approval and that of the members of our profession in this city and county—he will be well contented.

In conclusion, the writer desires to state in justice to his colleagues who have been members of the Board of Censors, that the charges preferred against members of this society for violation of the by-laws have not emanated from any of them nor from the chairman, but when such charges are made by other members, and the censors are appealed to by the members of the society for protection, it becomes the duty of the board to take notice of it and report the result of the investigation to the society. If the board is not sustained in the discharge of its duties and in its action, if members are allowed to violate the by-laws without being disciplined, if the dignity of the profession is not upheld, what use is there in having by-laws and a Board of Censors ? What is the object and purpose of the Medical Society of Erie County ?

Respectfully submitted for the Board of Censors.

EDWARD STORCK, *Chairman.*

Dr. J. B. Coakley moved that the report of the Board of Censors be adopted, together with the following:

*Resolved*, That the thanks of this society are due and are hereby tendered to Dr. Edward Storck for the faithful performance of his duties as Chairman of the Board of Censors. Carried.

The society then proceeded to the election of officers, with Drs. Edward Clark and J. J. Walsh acting as tellers. The election resulted as follows: *President*, William Warren Potter; *Vice-President*, William H. Gail; *Secretary*, Eli H. Long; *Assistant Secretary*, Ira C. Brown; *Treasurer*, Edward Clark; *Librarian*, Charles A. Ring; *Censors*, Edward Storck, H. R. Hopkins, P. W. Van Peyma, J. J. Walsh, Henry Lapp; *Membership Committee*, A. A. Hubbell, John A. Pettit, W. C. Callanan; *Delegates to the Medical Society of the State of New York*, B. H. Grove, Edward Clark, E. C. W. O'Brien, Ernest Wende, H. E. Hayd. The Treasurer was authorized to appoint his assistant officer.

The society then adjourned to meet at 2.45 p.m.

---

### AFTERNOON SESSION.

The society was called to order by the president at 3.15 P. M.

The president's address being in order, Dr. Coakley was called to the chair and Dr. O'Brien gave an address upon Progress in Medicine and Surgery, with special reference to work done in this country, this State and this County.

Under Miscellaneous Business, Dr. F. W. Abbott read an acknowledgment from Dr. Samo, which was ordered filed. Dr. Abbott moved that credit for dues be extended indefinitely to Dr. Samo. Carried.

The amendment to the by-laws offered by Dr. Storck at the last regular meeting was read, and in the absence of the mover was laid upon the table until the next regular meeting.

Upon a statement by the Secretary that our file of the Transactions of the Medical Society of the State of New York lack the volume for the year 1835, the officers were authorized to procure this volume if possible.

It was moved and carried that a committee of five be appointed to prepare a programme for the semi-annual meeting, three members to be appointed by the chair, the President and Secretary to be the other two. The chair appointed Drs. A. A. Hubbell, J. H. Pryor and H. E. Hayd.

By unanimous consent the President was authorized to appoint delegates to the Medical Association of Central New York.

The following resolution, moved by Dr. C.A. Ring and seconded by Dr. Putnam, was adopted :

*Resolved*, That our delegates to the Medical Society of the State of New York be instructed to request that Society by its Committee on Legislation to frame a bill for presentation to the Legislature, restricting the practice of hypnotism to legally qualified medical practitioners, and prohibiting public exhibitions of hypnotism.

A communication from the Secretary of the State Medical Society announcing its next meeting, etc., was received and filed.

The Secretary read a letter from Dr. Bernard Cohen, in which he expressed regret for the causes which led to his expulsion from membership, and asked to be reinstated. Upon motion of Dr. Hartwig, Dr. Cohen was reinstated by a unanimous vote.

The Secretary read letters of resignation of membership from Drs. J. Falk and G. H. McMichael.

Dr. B. H. Long moved that these resignations be laid on the table until an opinion was had of the Board of Censors as to whether a member's resignation can be accepted by the society.

Dr. Hubbell moved as a substitute that these resignations be accepted. Lost.

The motion to table was then carried. President O'Brien, Drs. Park, Putnam, and Hubbell found it necessary to be excused at this time.

The programme of scientific papers was then taken up and the following read: By Dr. M. D. Mann,—Laparatomy, Six Times in the Same Patient. By Dr. A. L. Benedict,—The Practical Use of the Metric System. This paper included a report from the committee appointed at the last meeting to report upon this subject at this time. The facts were presented that, as far as was ascertained, about fifteen of the physicians of this city employed the metric system exclusively in their prescriptions ; others use it to some extent ; it is used in the Buffalo General Hospital, the Marine ward in the Sisters of Charity Hospital, and, to some extent, in the Fitch Dispensary and other charitable institutions.

The committee asked that, in order to obtain more complete information, they be empowered to send a circular to each member of the society ; also that some formal action be taken endorsing the use of the metric system together with the apothecaries' system.

Upon motion the committee was authorized to issue such circular and the necessary appropriation for the purpose ordered.

Dr. Herman Mynter then read a paper entitled, Senile Gangrene. (To be published in a later issue of the Journal.)

The society then adjourned.

